# The efficacy of nasal administration of esketamine in patients having moderate-to-severe pain after preoperative CT-guided needle localization: a randomized, double-blind, placebo-controlled trial

**DOI:** 10.3389/fmed.2024.1344160

**Published:** 2024-04-09

**Authors:** Jiangning Xu, Jin Jian, Yunyun Zhang, Jingxiang Wu, Yuwei Qiu

**Affiliations:** ^1^Department of Anesthesiology, Shanghai Chest Hospital, Shanghai Jiao Tong University, School of Medicine, Shanghai, China; ^2^Department of Anesthesiology, The People’s Hospital of YuBei District, Chongqing, China; ^3^Outcomes Research Consortium, Cleveland, OH, United States

**Keywords:** pain, analgesia, pulmonary nodules, localization, video-assisted thoracoscopic surgery

## Abstract

**Background:**

Whether nasal administration of esketamine can provide effective analgesia is unclear in patients with acute pain after preoperative CT-guided needle localization.

**Methods:**

In this double-blind, randomized, placebo-controlled trial, patients were assigned to receive either nasal administration of esketamine (0.3 mg/kg or 0.5 mg/kg) or saline (identical in appearance to esketamine) when they had visual analog scale (VAS) pain scores >3/10 during deep breathing after preoperative CT-guided needle localization. The primary outcome was the percentage of patients with satisfactory pain relief, which was defined as VAS pain scores ≤3/10 measured 15 min after intranasal of esketamine or saline. Secondary outcomes included VAS measured following esketamine or saline, the incidence and cumulative dose of rescue hydromorphone use, and related adverse events.

**Results:**

A total of 90 patients were included in the final analysis. Following intranasal treatment, the percentage of patients with satisfactory pain relief was 16.7% (5/30) in the saline group, 56.7% (17/30) in the 0.3 mg/kg esketamine group, and 53.3% (16/30) in the 0.5 mg/kg esketamine group (*p* = 0.002). The median VAS during deep breathing was less after the intranasal administration of esketamine {median (IQR), 3 (3, 5) in 0.3 mg/kg or 0.5 mg/kg esketamine compared to the saline group [5 (4, 6)], *p* = 0.009}. The incidence of rescue hydromorphone use was detected less in the esketamine group compared to the saline group (43.3% in the 0.3 mg/kg esketamine group, 36.7% in the 0.5 mg/kg esketamine group, and 73.3% in the saline group, *p* = 0.010). The adverse events were similar among the three groups (*p* > 0.05).

**Conclusion:**

Intranasal administration of esketamine is easier and more effective in alleviating acute pain in patients after preoperative CT-guided needle localization without significant adverse effects.

## 1 Introduction

Low-dose computed tomography has detected millions of small pulmonary nodules (SPNs) and a substantial number of SPNs require resection by video-assisted thoracic surgery (VATS) (1). SPNs are difficult to palpate and finding a SPN during VATS without guidance can be fraught (2). Preoperative CT-guided needle localization can accurately localize SPNs prior to VATS, but needle localization may lead to substantial acute pain (3, 4). Our prospective observational study found that 50.8% of the patients had a visual analog scale (VAS) pain score ≥ 4 during deep breathing after preoperative needle localization (3). Consistently, another study showed that the localization-related pain score could reach 4.7 ± 1.6 (4). The substantial pain due to the rigid wire remaining in place would persist until surgical resection and it might greatly aggravate patients’ anxiety or fear prior to VATS (4, 5). Implementation of effective therapies is vital to solving this pain and enhancing patients’ satisfaction.

Several analgesic medications or regional blocks may alleviate the pain severity but they may also increase the burden of medical personnel resources, especially in developing countries. Finding a resource-less, relatively safe, and pain-sparing method is challenging for thoracic anesthesiologists. Ketamine, the N-methyl-D-aspartate receptor antagonist, is a potent analgesic without significant respiratory depression. Esketamine, the S-(+)-isomer of ketamine [S-(+)-K], has twice the analgesic potency compared to ketamine but with less psychomimetic side effects (6–8). The intranasal spray of esketamine has been approved by FDA in treatment-resistant depression (9, 10), but the analgesic feature of the intranasal spray of esketamine on acute pain has yet to be clarified. A previous study found that the intranasal spray of esketamine with midazolam was similar in effectiveness compared to standard morphine patient-controlled analgesia in the postoperative setting (11). Nonetheless, whether intranasal spray of esketamine alone could produce analgesic efficacy remains to be illustrated in patients after CT-guided needle localization.

Esketamine for intranasal delivery may provide more favorable mucosal absorption because of its relatively low molecular weight (12). The increased bioavailability of intranasal delivery may also lower the doses administered and thereby limit the adverse psychomimetic effects (13). Moreover, intranasal delivery of esketamine would circumvent the limitations associated with intravenous routes. Previous studies showed that esketamine’s effect was driven by its pharmacokinetics (14) and intranasal esketamine in patients with treatment-resistant depression existed in a dose-dependent manner (15). Whether intranasal administration of esketamine may have an ascending dose-dependent effect on acute pain remains controversial (15, 16).

In this study, we aimed to investigate whether intranasal administration of esketamine provides analgesia in patients receiving CT-guided needle localization. Specifically, we tested the primary hypothesis that the intranasal administration of esketamine increases the percentage of patients with satisfactory pain relief after CT-guided needle localization; Second, we tested the hypothesis that the intranasal administration of esketamine reduces the pain score, decreases the use of rescue analgesics, and does not increase adverse effects.

## 2 Materials and methods

### 2.1 Ethics and registration

This study protocol was approved by the Shanghai Chest Hospital Institutional Review Board (IRB IS22033), and written informed consent was obtained from each patient. This trial was registered before subject enrolment began at the Chinese Clinical Trial Registry (ChiCTR2200061734; principal investigator, Yuwei Qiu; date of registration, 1 July 2022).

### 2.2 Study design and participants

We conducted this randomized, controlled, and double-blinded clinical trial at Shanghai Chest Hospital. Eligible patients were between 18 and 75 years old, had an American Society of Anesthesiologists (ASA) physical status of I–III and body mass index between 18 and 30 kg/m^2^, diagnosed with SPNs requiring preoperative CT-guided needle localization, and had VAS pain score exceeding 3/10 during deep breathing after needle localization. Patients were excluded if they had clinically significant cardiovascular diseases, were unable to perform VAS, had chronic pain, including herpes zoster around chest regions and complex regional pain syndrome, or took opioids in the last month. Patients who had undergone previous thoracic surgeries were also excluded in case they might have neuropathic pain or intercostal nerve damage.

### 2.3 Randomization and masking

On the surgical day, the patients were admitted to the CT room for needle localization. After sterile prep and drape of the patient, 1% lidocaine was injected at the site of needle insertion by the radiologists. A Hawkins III Hardwire breast localization needle (20-gage, 12.5 cm in length) was then inserted through the chest wall and advanced to approach the small nodules.

Ten minutes after CT-guided needle localization, an investigating researcher assessed pain intensity using a 10 cm VAS (0 cm = no pain and 10 cm = worst imaginable pain) in the pre-anesthesia room. When VAS exceeded 3/10 cm during deep breathing, patients were randomized into one of the three groups using a set of computer-generated random numbers kept in sealed envelopes by an investigator not involved in clinical care. Envelopes were opened shortly before the medications were given to keep allocation concealed as long as practical. The three groups were: (1) intranasal spray of saline placebo; (2) intranasal spray of 0.3 mg/kg esketamine (Esketamine, Hengrui Medicine Co., Ltd., Jiangsu, China). 50 mg/2 mL; and (3) intranasal spray of 0.5 mg/kg esketamine. The pain assessors on site and patients were not informed of their group assignments.

### 2.4 Study drug and administration

**Figure 1:**
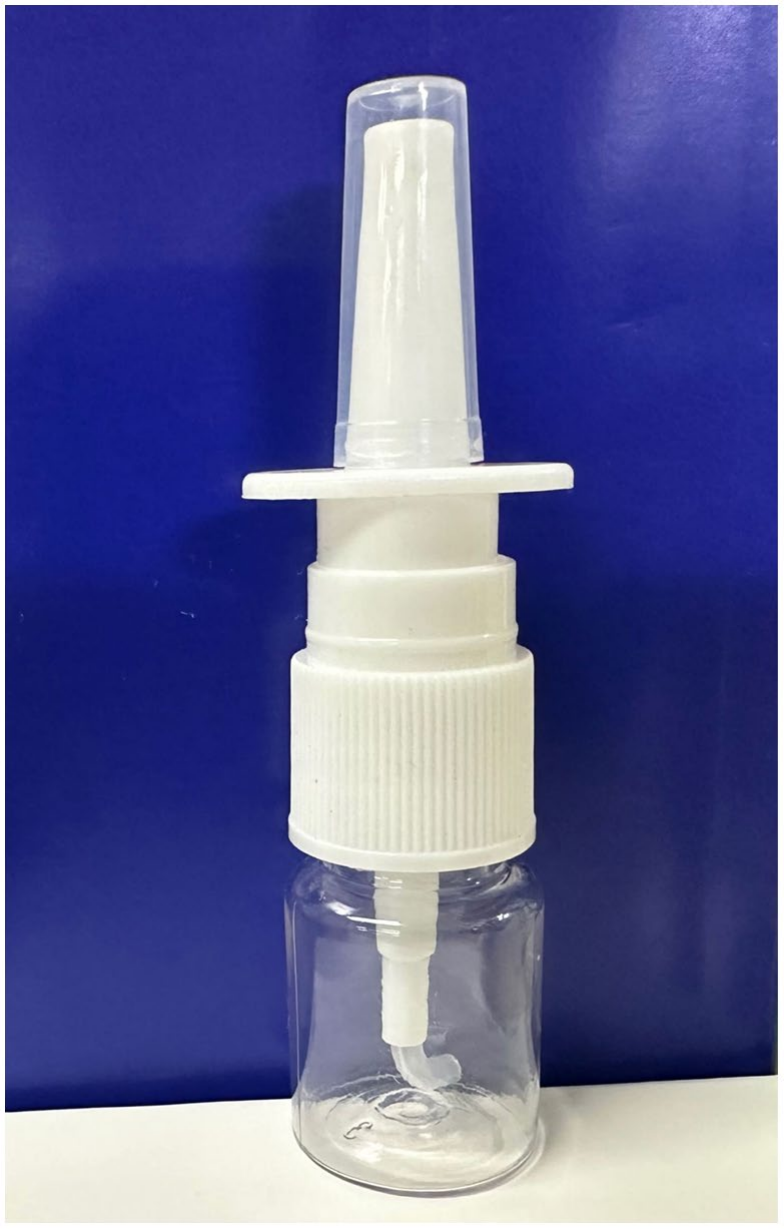
Nasal spray device. Study medication was provided in a disposable nasal spray device containing 1–2 mL of solution.

An independent investigator was in charge of the medication preparation according to the random sequence. Study medication was provided in a disposable nasal spray device containing 1–2 mL of either esketamine or placebo (i.e., 10–15 sprays, 100 μL/per spray, Figure 1). To maintain blinding, the placebo (intranasal solution of saline) was prepared identical in appearance to esketamine. After the patients signed the written informed consent, they were given esketamine or saline placebo into each nostril at different points, each separated by 10 s.

### 2.5 Outcome assessments

Analgesic efficacy was assessed at 5 min, 10 min, and 15 min after esketamine or saline was given, using VAS, by the pain assessors who were blinded to group assignments. The primary outcome was the percentage of patients with satisfactory pain relief, which was defined as VAS pain scores ≤3/10 during deep breathing measured 15 min after intranasal of esketamine or saline. If VAS pain scores still exceeded 3/10 at 15 min after nasal spray, the nurses started to establish intravenous access, and rescue hydromorphone was given from 0.5 mg to 2 mg at intervals until VAS ≤ 3. Then, adverse effects were assessed at 5 min after hydromorphone use.

Secondary outcomes included the VAS pain score, incidence and cumulative dose of hydromorphone use, and adverse events. Adverse events included dizziness, over-sedation, hallucinations, nausea, vomiting, confusion and disorientation, and rashes during the 15-min period after study medication was given. Sedation is assessed by the Richmond Agitation Sedation Scale (RASS) (17), which is a 10-point scale from −5 to +4, with −5 denoting not responding to voice or physical stimulation and + 4 denoting combative or violent. We also assessed the pain intensity in the post-anesthesia care unit (PACU).

### 2.6 Statistical analysis

#### 2.6.1 Sample size estimation

We conducted a pilot study and found the percentage of patients having VAS pain score ≥ 3/10 was 60% in 0.3 mg/kg esketamine nasal administration (6/10) and 80% in saline placebo (8/10), respectively. Accounting for the potential dose-dependent profile, we assumed that increasing the dose to 0.5 mg/kg may further reduce the percentage of moderate-to-severe pain to 30%. The effect size was then calculated as 0.41 by PASS 15.0, and a sample size of 75 patients had 90% power to detect a 5% two-sided significance. To account for 10% of dropouts, we increased the sample size to 90 (30 subjects per group).

#### 2.6.2 Data analysis

Continuous or discrete data were described using mean and standard deviation (SD) or median and 25th and 75th quartiles. Categorical data were described using numbers and percentages.

Kolmogorov–Smirnov test was used to test whether the continuous variables met the normal distribution. F-test was used to compare the effects on normally distributed continuous outcomes. Otherwise, the Mann–Whitney U-test was used when continuous outcomes or data were skewed or met the non-normal distribution.

Primary efficacy analyses were performed in the intention-to-treat population of all randomized patients. The percentage of moderate-to-severe pain, adverse events, and hydromorphone use was compared between the three groups using χ2 test or Fisher’s exact test. Each median score of VAS before the nasal administration and 5 min, 10 min, and 15 min after the nasal administration was compared using the Mann–Whitney U-test.

Statistical analyses were performed with SPSS (version 25, IBM Statistics, United States). All reported *p*-values were two-sided, and a p-value under 0.05 was considered statistically significant.

## 3 Results

### 3.1 Patients

**Figure 2:**
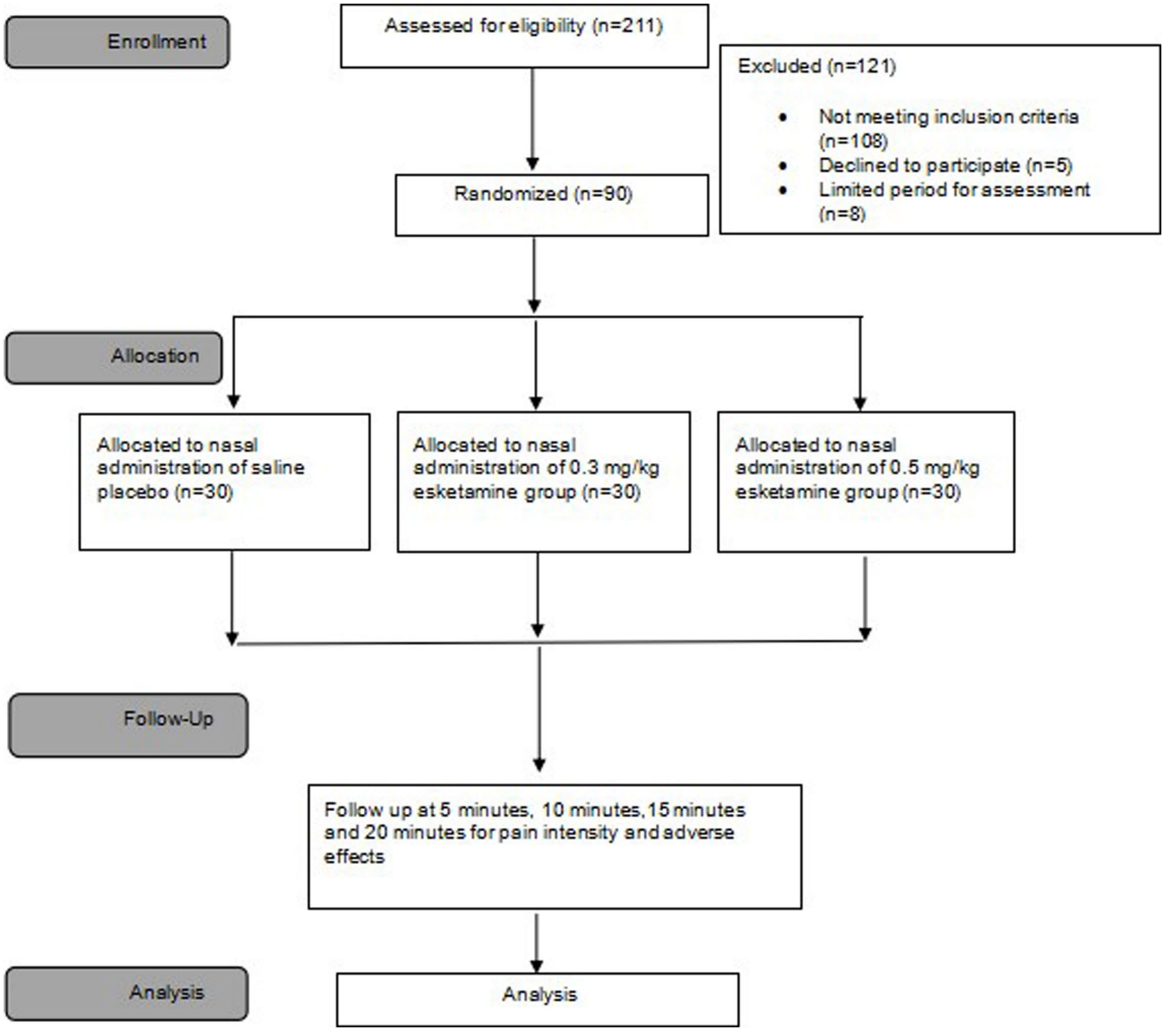
Flow diagram of subjects’ recruitment and treatment.

From 5 May 2022 to 20 December 2022, we screened a total of 211 patients diagnosed with SPNs requiring preoperative CT-guided needle localization. Finally, 90 patients with VAS exceeding 3/10 during deep breathing after needle localization were randomly assigned to receive either of the intended interventions. All 90 patients were included in the final analysis (Figure 2). Patient characteristics are summarized in Table 1. The mean age was 54 (SD = 12) years and 71.1% were female. Baseline characteristics and needle-location data were comparable between groups (Table 1).

**Table 1:** Baseline, demographics, and needle location-related factors.

Variables	Nasal saline placebo *n* = 30	Nasal 0.3 mg/kg esketamine *n* = 30	Nasal 0.5 mg/kg esketamine *n* = 30	*p*-value
Age, year	53 ± 12	55 ± 13	53 ± 10	0.696
Gender Female, *n* (%)	21 (70)	21 (70)	22 (73.3)	0.947
Height, cm	164.80 ± 8.03	164.27 ± 6.53	162.70 ± 7.13	0.510
Weight, kg	61.17 ± 11.87	59.43 ± 9.39	60.63 ± 9.19	0.798
BMI, kg/m^2^	22.39 ± 3.15	21.97 ± 2.78	22.87 ± 2.98	0.499
ASA status, %				0.517
I	2 (6.7)	4 (13.3)	2 (6.7)	
II	16 (53.3)	18 (60)	21 (70)	
III	12 (40)	8 (26.7)	7 (23.3)	
Education level				0.551
Middle school	13 (43.3)	14 (46.7)	10 (33.3)	
High school and above	17 (56.7)	16 (53.3)	20 (66.7)	
Number of Needles	1 [1–2]	1.5 [1–2]	1 [1–2]	0.261

Data are presented as means ± SDs, median (inter-quartile range, 25th percentile–75th percentile) or number (%). F-test was used for normally distributed continuous outcomes, Mann–Whitney U-test for non-normally distributed outcomes, and chi-square or Fisher’s exact tests for category outcomes. BMI, body mass index; ASA, American Society of Anesthesiologists; VAS, visual analog pain scores. Moderate-to-severe pain is defined when VAS pain scores exceeded 3/10.

### 3.2 Primary outcome

Before the nasal administration of study drugs, VAS during deep breathing was 5 [5,7] in the saline group, 5.5 [5,7] in the 0.3 mg/kg esketamine group, and 5 [4,7] in the 0.5 mg/kg esketamine group, respectively (*p* > 0.05, Table 1). After nasal administration of 0.3 mg/kg or 0.5 mg/kg esketamine, pain intensity reduced in a time-dependent manner (*p* = 0.05, Table 2). At 15 min after the nasal administration of study drugs, the percentage of patients having VAS exceeding 3/10 during deep breathing was 83.3% in the saline group, 43.3% in the 0.3 mg/kg esketamine group, and 46.7% in the 0.5 mg/kg esketamine group, respectively (*p* = 0.002, Table 2).

**Table 2:** Efficacy outcomes of nasal administration of saline, 0.3 mg/kg esketamine, and 0.5 mg/kg esketamine on acute pain after CT-guided needle localization.

Pain parameters	Nasal saline placebo *n* = 30	Nasal 0.3 mg/kg esketamine *n* = 30	Nasal 0.5 mg/kg esketamine *n* = 30	*p*-value
Incidence of moderate-to-severe pain after treatment during deep breathing, *n* (%)	25 (83.3)	13 (43.3)	14 (46.7)	0.002**
Absolute median VAS reduction (15 min minus before treatment)	1 [0–2]	2 [1–2]	2 [1–3]	0.024*
VAS at rest before treatment	3.5 [2–5.25]	4.5 [3–6]	4 [3–5]	0.411
VAS during deep breathing before treatment	5 [5–7]	5.5 [5–7]	5 [4–7]	1.000
VAS at 5 min after treatment during deep breathing	5.5 [4.75–6.0]	4.5 [3–7]	5 [4–6.0]	0.480
VAS at 10 min after treatment during deep breathing	5 [4–6.25]	4 [3–5.25]	4 [3–6.0]	0.148
VAS at 15 min after treatment during deep breathing	5 [4–6]	3 [3–5]	3 [3–5]	0.009**
VAS at 15 min after treatment at rest	3 [2–4]	2 [1–3.25]	2.5 [2–3]	0.875
Incidence of rescue hydromorphone use, *n* (%)	22 (73.3)	13 (43.3)	11 (36.7)	0.010*
Cumulative dose of hydromorphone use, mg	1 [0–1]	0 [0–1]	0 [0–1]	0.012*
VAS at rest in PACU	2 [0–2]	2 [1–2]	1 [0–2]	0.328
VAS during breathing in PACU	3 [2–4]	3 [2–4]	3 [2–4]	0.669

Data are presented as median (inter-quartile range, 25th percentile–75th percentile), or number (%). Chi-square or Fisher’s exact tests for binary outcomes. Mann–Whitney U-test for non-normally distributed outcomes. Denotes statistically significant (*p < 0.05; **p < 0.01) differences among groups.

### 3.3 Secondary outcomes

Both doses of nasal esketamine reduced the pain intensity compared to saline placebo (Table 2). The median VAS during deep breathing was significantly less in nasal esketamine groups than in saline placebo at 15 min after administration (*p* = 0.009, Table 2), but the pain scores did not differ between the two doses of esketamine (*p* > 0.05, Table 2).

Patients given either nasal esketamine or rescue hydromorphone had less use of rescue hydromorphone than the saline group, with 73.3% (22/30) in the saline group compared to 43.3% (13/30) in 0.3 mg/kg esketamine and 36.7% (11/30) in 0.5 mg/kg esketamine (*p* = 0.01, Table 2). The cumulative dose of hydromorphone decreased from the median dose of 1 mg in the saline group to 0 mg in both esketamine groups (*p* = 0.012, Table 2). After rescue hydromorphone use, more patients experienced dizziness in the saline group than in both esketamine groups (*p* = 0.015, Table 3).

**Table 3:** Adverse events in the study participants.

Adverse effects	Nasal saline placebo *n* = 30	Nasal 0.3 mg/kg esketamine *n* = 30	Nasal 0.5 mg/kg esketamine *n* = 30	*p*-value
After nasal administration				
Nausea, *n* (%)	0 (0)	0 (0)	2 (6.7)	0.326
Vomiting, *n* (%)	0 (0)	0 (0)	1 (3.3)	1.000
Rash, *n* (%)	0 (0)	0 (0)	1 (3.3)	1.000
Dizziness, *n* (%)	0 (0)	4 (13.3)	5 (16.7)	0.071
Confusion and disorientation, *n* (%)	0 (0)	0 (0)	0 (0)	Not applicable
Hallucinations, *n* (%)	0 (0)	0 (0)	0 (0)	Not applicable
Desaturation, *n* (%)	1 (3.3)	0 (0)	1 (3.3)	1.000
RASS	0 [0–0]	0 [0–0]	0 [0–0]	Not applicable
After rescue hydromorphone use, *n* (%)				
Dizziness, *n* (%)	13 (43.3)	8 (26.7)	3 (10)	0.015*
Drowsiness, *n* (%)	5 (16.7)	1 (3.3)	5 (16.7)	0.215

Data are presented as numbers (%). Abbreviations: RASS, Richmond Agitation Sedation. Scale. Denotes statistically significant (*p < 0.05; **p < 0.01) differences among groups.

### 3.4 Adverse effects

The incidence of nausea, vomiting, rash, dizziness, and desaturation was not statistically different among the three groups (*p* > 0.05, Table 3). Nonetheless, we found the incidence of dizziness increased after esketamine administration, from 0 in the saline group to 13.3% in 0.3 mg/kg esketamine and 16.7% in 0.5 mg/kg esketamine, even without reaching a statistical difference (*p* > 0.05, Table 3). None of the patients experienced confusion, disorientation or hallucinations. The pain intensity in PACU was similar among the three groups (*p* > 0.05, Table 2).

## 4 Discussion

In patients after preoperative CT-guided needle localization, intranasal administration of either 0.3 mg/kg or 0.5 mg/kg esketamine reduced the incidence of moderate-to-severe pain by half compared with nasal saline. In parallel, the VAS pain score was also reduced from the median VAS at 5 in the saline group to 3 in both nasal esketamine groups. Due to the alleviation of moderate-to-severe pain after nasal esketamine, rescue hydromorphone use and related adverse effects were greatly reduced. Therefore, intranasal esketamine provided a feasible and resource-sparing route of analgesic delivery in the preoperative acute pain setting.

The localization needle passed through the skin, penetrated the lung parietal and visceral parenchyma, anchored the pulmonary nodules, and then kept the rigid wire in place until resection (18, 19). Hence, the needle localization-related pain may be sustained until surgical resection. Timely analgesia is critical for those patients with moderate-to-severe pain before VATS. The substantial reduction in the incidence of moderate-to-severe pain (approximately one-half) and pain intensity after the nasal spray of esketamine is clinically meaningful in the preoperative pain setting. Nasal administration of esketamine may thus serve as a possible therapeutic measure for acute pain after CT-guided needle localization. Although intranasal esketamine was approved by the FDA for treatment-resistant depression (9, 20, 21), the evidence testing intranasal esketamine for acute pain was scarce (11, 22, 23). A pilot study including 22 patients found that intranasal spray of esketamine combined with midazolam was similar in analgesic effectiveness compared to standard morphine patient-controlled analgesia in patients after spine surgery (11). Intranasal spray of esketamine could thus be considered a non-invasive analgesic alternative in patients with challenging IV access. A recent trial showed esketamine nasal drops in children after tonsillectomy could reduce pain and shorten the recovery time (23). Our hypothesis was inherited from the pilot study (11), and our data were similar to the two above studies (11, 23) that the intranasal spray of esketamine could be used as a non-invasive analgesic to alleviate acute pain in the pre-anesthesia setting.

There is no evidence supporting which single intranasal dose should be chosen for acute pain related to needle localization in adult patients. We selected 0.3 mg/kg or 0.5 mg/kg doses according to existing literature and our assumptions. Recently, several studies showed that small doses of esketamine might be enough to reduce pain scores (24). A dose of 0.2 mg/kg IV esketamine before the induction of anesthesia was recommended to reduce the pain of propofol injection (6, 24). Another trial showed that intravenous injection of 0.25 mg/kg esketamine improved pain during exercise at 24 h post-operatively in patients receiving elective cesarean delivery (25). Subanesthetic doses of esketamine reduced postoperative pain in patients scheduled for laparoscopic cholecystectomy in the PACU (26). The analgesic effects of intranasal esketamine were supposed to be mediated by being absorbed through the nasal cavity, and a previous study showed that the dose of esketamine absorbed through the nasal cavity was reduced by 38% after 28-mg dose (13), which meant 60% of esketamine or higher dose might be intravenously absorbed. According to the quantified absolute nasal bioavailability of esketamine, we chose 0.3 mg/kg intranasal esketamine (approximately 0.2 mg/kg intravenously) as the potential effective dose. We also wanted to test whether there was a dose-dependent manner of nasal esketamine, so we selected a 0.5 mg/kg nasal dose (approximately 0.3 mg/kg intravenous dose). Our result demonstrated that 0.3 mg/kg intranasal esketamine was clinically effective in reducing moderate-to-severe pain during breathing. Nonetheless, our data did not support the dose-dependent manner of intranasal esketamine. Consistent with Brinck’s findings (16), we did not detect a difference in pain relief between 0.3 mg/kg and 0.5 mg/kg nasal esketamine. Higher nasal esketamine could not improve pain alleviation further but might increase the risk of dizziness.

There are several ways to treat localization-related pain. Lidocaine topical infiltration around the insertion site yielded optimal pain control, as we found previously (3). We assumed that the parietal pleura-induced pain is perhaps dominant and may hardly be relieved by non-steroidal anti-inflammatory drugs (27, 28). The parietal pain is mainly supplied by the intercostal nerves on its lateral aspects, by the T1 spinal nerve on its apex, and by the phrenic nerves on the diaphragm (29). Intercostal, interpleural, epidural, and paravertebral blocks have all been proven useful in controlling pleuritic pain (29, 30), but all blocks will consume medical resources and perhaps lead to some complications. Potent opioids are historically effective in relieving significant pain but may have adverse effects. As we found in our study, rescue hydromorphone use led to more than 40% of patients experiencing dizziness in the saline group. We proved that 0.3 mg/kg nasal esketamine could serve as a resource-sparing and non-invasive method to treat needle-location pain without opioid-related side effects.

We also wanted to verify the analgesic effectiveness of nasal esketamine was timely, so we designed 15 min as the therapeutic window. A previous study showed the fraction of the esketamine dose absorbed through the nasal cavity was complete and fast, and the mean absorption time was 0.341 h, as previously reported (16). We observed the analgesic effect of esketamine from the start of nasal administration until 15 min at 5-min intervals. The nasal esketamine showed a time-dependent manner of analgesia, and 0.3 mg/kg or 0.5 mg/kg of nasal esketamine demonstrated effectiveness in pain relief after 15 min.

This trial has several limitations. We conducted the trial in a single tertiary center, and the results needed to be verified in more generalized institutions. Second, we set two fixed doses to investigate in a dose-dependent manner. Although the chosen doses depended on the population pharmacokinetics of esketamine nasal spray in healthy subjects and previous pain studies, the linear or proportional dose-effect manner should be investigated further. Third, we set a saline nasal group to mask the positive drug and mimic the placebo effect, but we did not add the bittering agent to the intranasal placebo to simulate the taste of the esketamine solutions, which might affect patients’ objective self-assessment. Fourth, our sample size was designed to test the analgesic effect of esketamine on moderate-to-severe pain, but the sample size was not enough to differentiate the effect on moderate pain or severe pain, respectively. Finally, we did not observe the effect of esketamine after 15 min, which would underestimate the analgesic effect of esketamine.

## 5 Conclusion

In patients after preoperative CT-guided needle localization, nasal administration of 0.3 mg/kg or 0.5 mg/kg esketamine could reduce the incidence of moderate-to-severe pain by half compared with saline. Nasal spray of esketamine could be used as a feasible and non-invasive method to alleviate acute thoracic pain in the pre-anesthesia setting.

## Data availability statement

The raw data supporting the conclusions of this article will be made available by the authors, without undue reservation.

## Ethics statement

The studies involving humans were approved by Shanghai Chest Hospital Institutional Review Board. The studies were conducted in accordance with the local legislation and institutional requirements. The participants provided their written informed consent to participate in this study.

## Author contributions

JX: Data curation, Formal analysis, Investigation, Writing – review & editing. JJ: Data curation, Formal analysis, Investigation, Writing – review & editing. YZ: Investigation, Resources, Writing – review & editing. JW: Conceptualization, Supervision, Methodology, Writing – review & editing. YQ: Conceptualization, Funding acquisition, Methodology, Project administration, Supervision, Validation, Writing – original draft, Writing – review & editing, Investigation.
